# Decolorization and COD removal from real textile wastewater by chemical and electrochemical Fenton processes: a comparative study

**DOI:** 10.1186/2052-336X-11-31

**Published:** 2013-12-19

**Authors:** Akbar Eslami, Mahsa Moradi, Farshid Ghanbari, Fayyaz Mehdipour

**Affiliations:** 1 Department of Environmental Health Engineering, School of Public Health, Shahid Beheshti University of Medical Sciences, Tehran, Iran; 2 Department of Environmental Health Engineering, School of Public Health, Ahvaz Jundishapur University of Medical Sciences, Ahvaz, Iran

**Keywords:** Chemical Fenton, Decolorization, Electrochemical Fenton, Textile wastewater

## Abstract

**Background:**

Due to the presence of non-biodegradable and toxic compounds, textile wastewater is difficult to treat by conventional methods. In the present study, Electrochemical Fenton (EF) and Chemical Fenton (CF) processes were studied and compared for the treatment of real textile wastewater. The effects of electrical current, ferrous ion, hydrogen peroxide concentration and reaction time on the removal efficiencies of COD and color were investigated. All the experiments were carried out at pH = 3.

**Results:**

Both EF and CF processes were mostly efficient within hydrogen peroxide concentration of 1978 mg/L (H_2_O_2_: COD ~ 1.1). The highest COD and color removal efficiencies were 70.6% and 72.9% respectively which were obtained through the EF process in 350 mA electrical current, 1978 mg/L hydrogen peroxide and 60 minutes reaction time. Furthermore, the operational costs of EF and CF processes were 17.56 and 8.6 US$ per kilogram of the removed COD respectively.

**Conclusion:**

It was concluded that the electrochemical Fenton process was more efficient than the chemical Fenton process in the degradation of textile wastewater. Likewise, Although EF process imposed higher operational costs than the CF; it dramatically decreased the reaction time to gain the highest degradation efficiency.

## Introduction

Textile industry is one of the largest consumers of water (800–1000 m^3^/ton), and consequently, one of the largest producers of wastewater among all industries [1,2]. Textile wastewater from dyeing and finishing processes has been a serious environmental threat for years [3]. This wastewater, with a Chemical Oxygen Demand (COD) concentration exceeding 1600 mg/L and a strong dark color, is classified as a high strength wastewater [4]. Moreover, textile wastewaters exhibit low BOD to COD ratios (< 0.1) indicating their non-biodegradable nature [5]. The discharge of this type of wastewater without any treatment brings about considerable adverse impacts on the receiving water bodies crying out for an efficient treatment process [2]. Textile wastewater is usually treated by conventional methods such as biological oxidation [3,6], chemical coagulation and activated carbon adsorption [3]. However, these conventional methods suffer from a few limitations or drawbacks related to cost, efficiency and sludge generation [6]. The limitations of conventional methods can be overcome by the Advanced Oxidation Processes (AOPs). AOPs are promising alternatives which can produce hydroxyl radicals (^•^OH) and have been proven to be an efficient method for the degradation of the dyes and refractory pollutants [7,8]. The hydroxyl radical is a nonselective and extremely powerful oxidant that rate constants with organic matters fall in to a range of 10^9^ - 10^10^ M^-1^S^-1^[9-11]. Among the AOPs, Chemical Fenton (CF) process is particularly attractive for its simplicity without requirement for special equipment [12] and also for its ability in partial mineralization, lowering toxicity, and increasing wastewater susceptibility to biodegradation [13]. During CF process, ferrous ion reacts with hydrogen peroxide to generate the hydroxyl radical at low pH [14-16]:

(1) $${\mathrm{Fe}}^{2+}+H_{2}O_{2}\to {\mathrm{Fe}}^{3+}{+}^{•}\mathrm{OH}+{\mathrm{HO}}^{-}$$

CF process essentially depends on pH, temperature, hydrogen peroxide and ferrous ion concentrations and chemical structure of the organic compounds [17]. In recent years, there has also been an increasing interest in the use of electrochemical methods for the destruction of toxic and biorefractory organic pollutants [18]. Electrochemical methods are environmental-friendly technologies in environmental remediation as the main reagent used is electron, which is a clean reagent and therefore there is no need for adding reagent [19].

Nowadays, amongst hybrid processes, electrochemical Fenton, photo-Fenton, and sono-Fenton processes are mostly applied to increase the efficiency of Fenton process [20,21]. Particularly, electrochemical Fenton process has been studied by Kurt et al. (2007) and Atmaca (2009) for treatment of tannery wastewater and landfill leachate respectively achieving considerably good results [20,22]. Electrochemical Fenton (EF) processes include electrochemical reactions that generate one or both of the Fenton's reagents in situ. In the present study, within EF process, ferrous ion was generated by sacrificial anode (Iron) and H_2_O_2_ was introduced from out site. In this type of EF process, ferrous ion is generated continuously in the electrochemical cell that is also responsible for electrochemical coagulation of organic matters [22,23]:

(2) $${\mathrm{Fe}}_{\left(s\right)}\to {\mathrm{Fe}}^{2+}{}_{\left(\mathrm{aq}\right)}+{2e}^{-}$$

Most of the previous researches have been focused on the electrocoagulation and Fenton oxidation separately [2,4]. Also, there have not been wide studies which compare Fenton process with electrochemical Fenton process on the real textile wastewater and most studies were carried out on synthetic dyes as simple model of simulated textile wastewater [7,12]. The decolorization and COD removal from the real textile wastewater by CF and EF processes have not been studied in the literatures as a comparative study.

The aim of the present work was mainly to investigate the efficiency of the electrochemical Fenton and chemical Fenton processes for the removal of color and COD from a real textile wastewater. Besides, a comparison of the two mentioned processes was made considering their degradation efficiencies, the experimental conditions in which the processes had their highest efficiencies and their operational costs.

## Materials and methods

### Chemicals and reagents

Real textile wastewater sample, whose characteristics are illustrated in Table 1, was obtained from a textile plant located in Zanjan, Iran. The sample was kept in a dark container with a constant temperature of 4°C, without adding any chemical. Hydrogen peroxide (35%, w:w), iron (II) sulfate heptahydrate (FeSO_4_.7H_2_O), sulfuric acid and sodium hydroxide were purchased from Merck. In addition, potassium dichromate, silver sulfate and mercury sulfate were supplied from Fluka, Sigma Aldrich and Acros organics respectively.

**Table 1:** Real textile wastewater sample characteristics

**Parameter**	**Unit**	**Value**
COD	mg/L	1800
BOD_5_	mg/L	320
Absorbance (λ_max_ = 400 nm)	cm^-1^	1.036
Color	ADMI	1080
EC	mS/cm	0.50
pH	------	6.50

### Experimental apparatus

**Figure 1:**
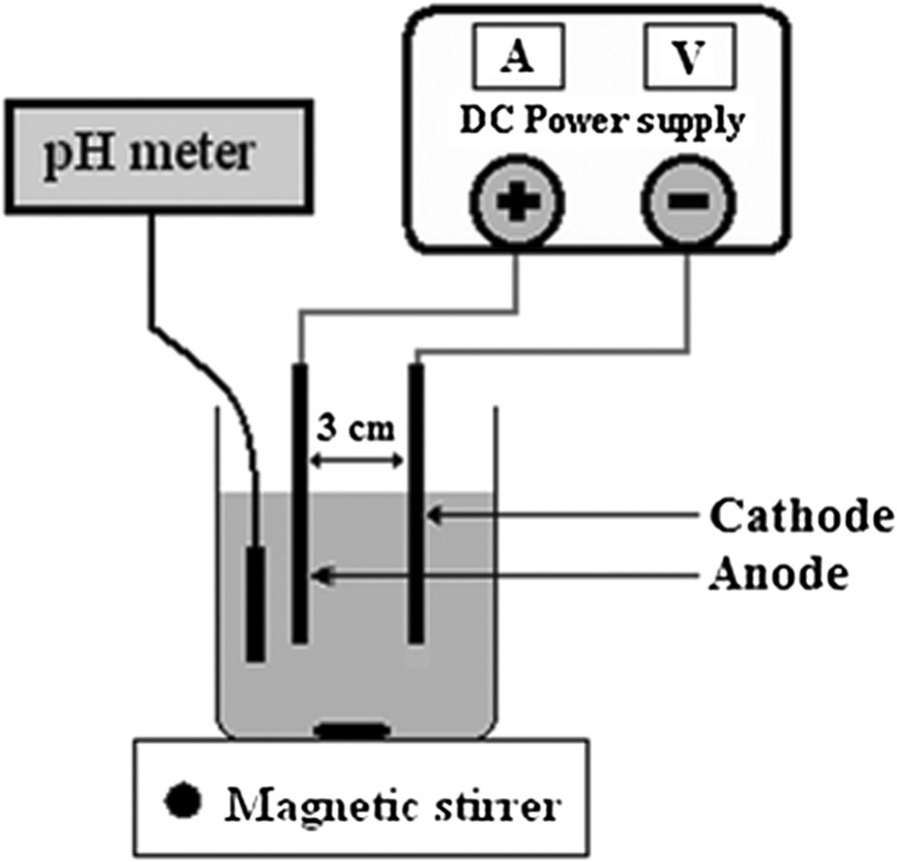
Scheme of the EF experimental setup.

The experimental setup is schematically shown in Figure 1. Electrochemical Fenton process consisted of an undivided reactor, with a volume of 250 ml, containing 200 ml sample. A magnetic stirrer was used to provide sufficient mixing inside the reactor. A pair of iron electrodes, having a dimension of 10 × 4 × 0.1 cm, with a distance of 3 cm, was applied as anode and cathode connecting to a digital DC power supply (Zhaoxin, 0.00-5 A, and 0.0-60 V). The total effective surface area of the electrodes was 40 cm^2^.

### Experimental procedure

#### Electrochemical Fenton process

The experiments were carried out at room temperature. As the optimal pH value recognized for the Fenton oxidation is around 3, the initial pH of the solution was adjusted to 3 by adding appropriate amount of sulfuric acid [17]. Then in each run of the experiments, hydrogen peroxide in different concentrations was applied dropwise with the electrical currents of 150, 250, 350 and 450 mA for each of the concentrations. Samples were taken in each reaction time as the process was terminated by turning the DC power supply off. In the next step, for the formation of Fe(OH)_3_, pH of the solution was adjusted to 9 by adding 4 N NaOH and was allowed to settle for 1 hour. Then the supernatant was withdrawn, heated in a 50°C water bath for 30 minutes to remove any residual hydrogen peroxide from the solution [24] and filtered through 0.45 μm.

#### Chemical Fenton process

CF experiments were performed with the same laboratory-made apparatus used for the EF process, except the iron electrodes and DC power supply. Adjusting the pH to 3, in order to add ferrous ion, FeSO_4_.7H_2_O was used in concentrations of 50, 150, 250, 350 mg/L Fe^2+^.

#### Analytical methods

The pH value was measured by a pH meter (WTW 720). A conductivity meter (HACH) was used to determine EC. Likewise, the color values were obtained by the ADMI tristimulus filter method via a HACH spectrophotometer UV–vis DR 5000 [25]. COD measurements were carried out using high range COD ampoules (HACH Chemical) with a spectrophotometer (DR 5000, HACH). The concentrations of other parameters of the wastewater were measured according to the standard methods [25]. Percentage of decolorization was calculated as follows:

(3) $$\mathrm{Decolorization}=\left(1-\frac{\mathrm{ADMI}}{\mathrm{ADM}I_{0}}\right)\times 100$$

Where ADMI_0_ and ADMI stand for the initial and final color of the solution respectively.

## Results

### Electrochemical Fenton process

#### Effect of electrical current

**Figure 2:**
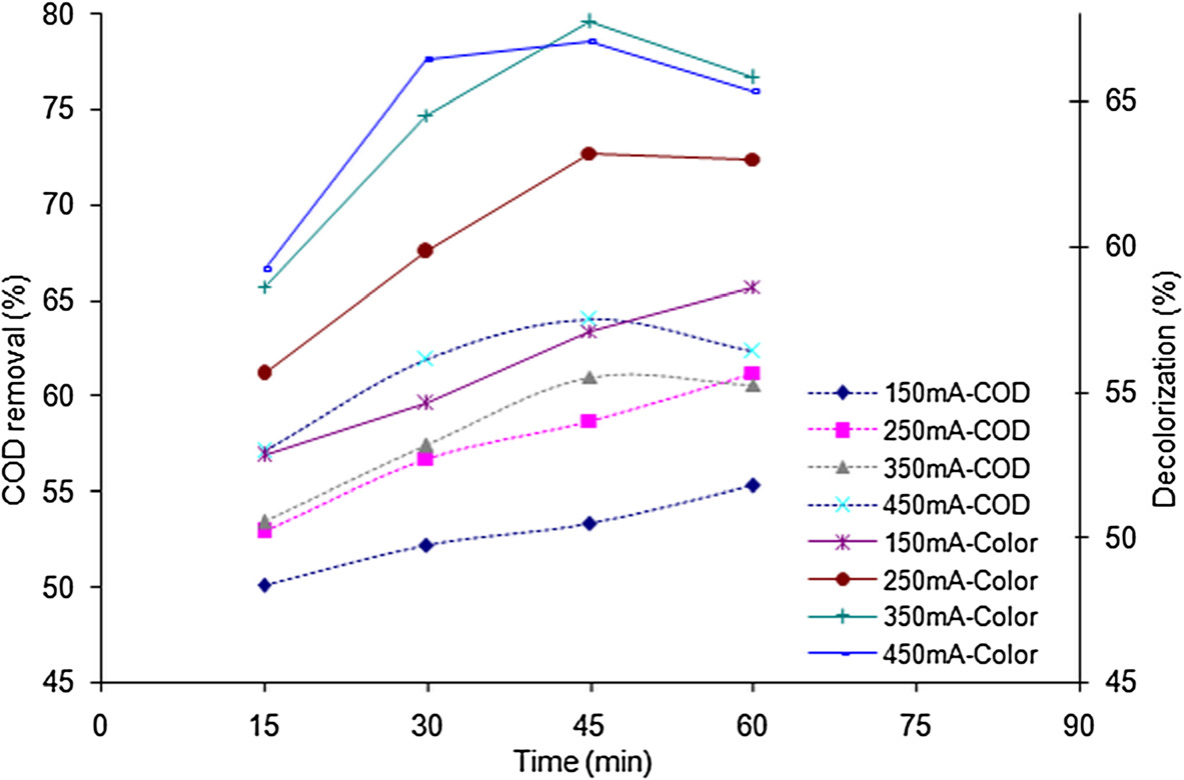
**Effects of electrical current and reaction time on the efficiencies of COD removal and decolorization; ;H**_**2**_**O**_**2**_ **= 1483 mg/L, pH = 3.**

**Figure 3:**
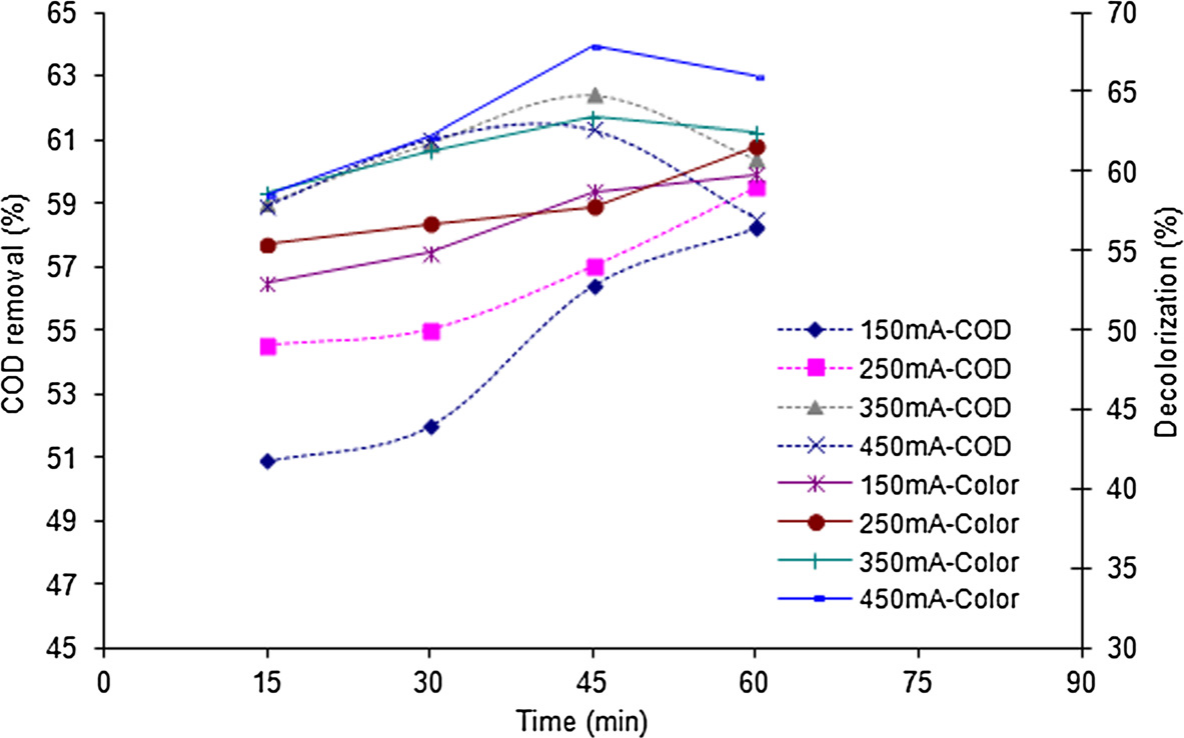
**Effects of electrical current and reaction time on the efficiencies of COD removal and decolorization; H**_**2**_**O**_**2**_ **= 2472 mg/L, pH = 3.**

In order to investigate the optimum applied current of the process, various electrical currents ranging from 150 to 450 mA were tested and the corresponded effects on COD removal and decolorization were studied. The results are depicted in Figures 2 and 3.

#### Effect of H_2_O_2_ concentration

**Figure 4:**
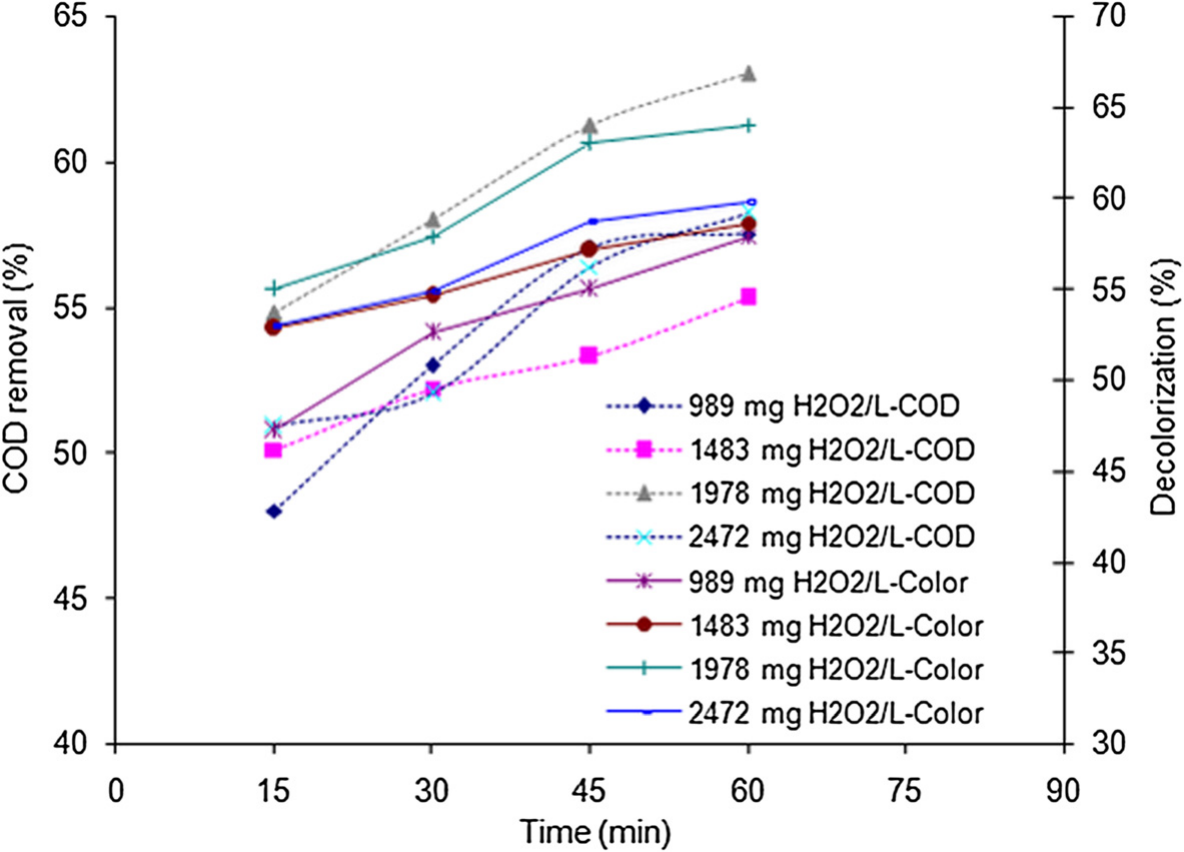
Effects of hydrogen peroxide concentration and reaction time on COD and color removal efficiencies; electrical current: 150 mA, pH = 3.

**Figure 5:**
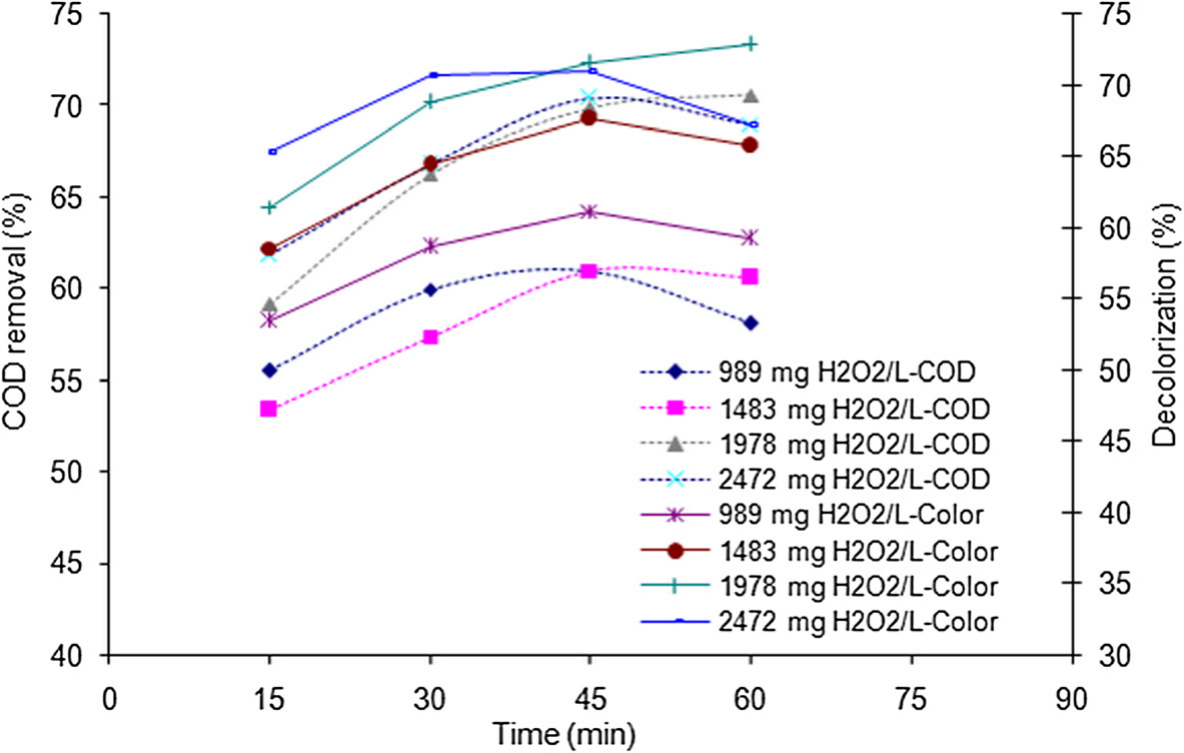
Effects of hydrogen peroxide concentration and reaction time on COD and color removal efficiencies; electrical current: 350 mA, pH = 3.

The effect of hydrogen peroxide concentration on the removal of COD and color was investigated and the results are illustrated in Figures 4 and 5.

#### Chemical Fenton process

**Figure 6:**
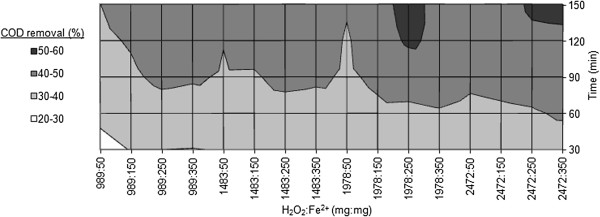
**Effects of H**_**2**_**O**_**2**_**:Fe**^**2+ **^**and reaction time on COD removal efficiency within CF process (pH = 3).**

**Figure 7:**
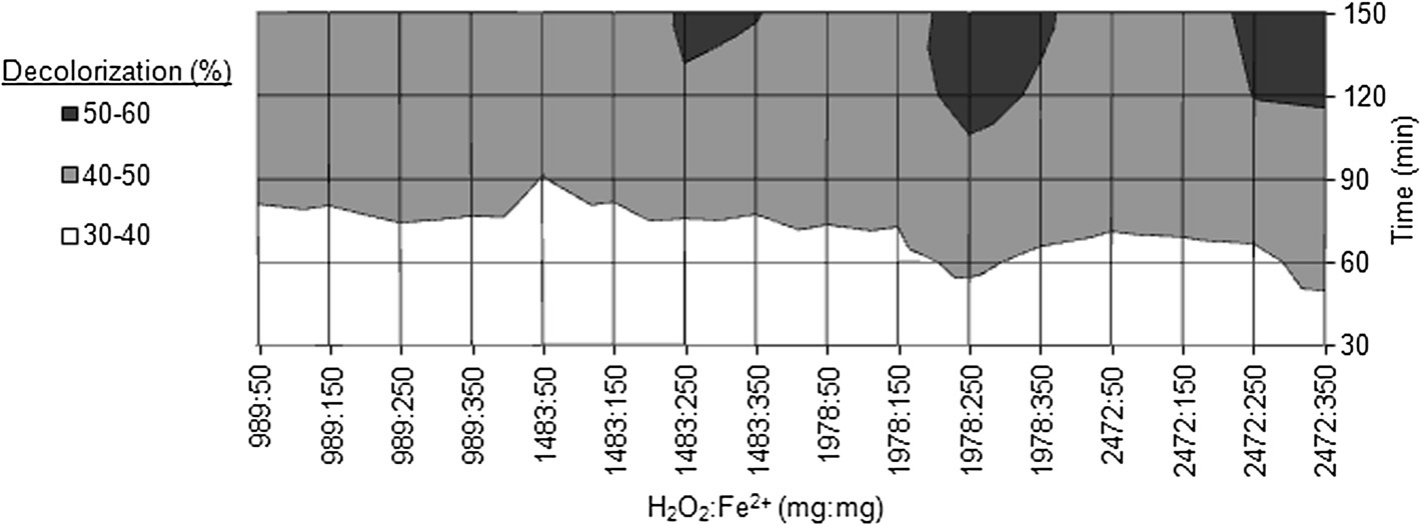
**Effects of H**_**2**_**O**_**2**_**:Fe**^**2+ **^**and reaction time on decolorization efficiency within CF process (pH = 3).**

The effect of hydrogen peroxide and ferrous ion ratio within various oxidation times on COD removal and decolorization are evaluated and the results are demonstrated in Figures 6 and 7 respectively. The concentrations of hydrogen peroxide applied for the CF process are the same as that of the EF process.

#### Comparison of electrochemical Fenton and chemical Fenton processes

**Figure 8:**
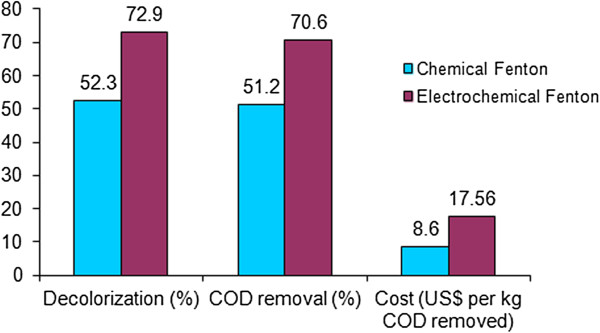
**Comparison of CF and EF efficiencies for the removal of COD and color and their operational costs; (CF: H**_**2**_**O**_**2**_ **= 1978 mg/L, Fe**^**2+**^ **= 250 mg/L, reaction time = 120 min) - (EF: H**_**2**_**O**_**2**_ **= 1978 mg/L, electrical current = 350 mA, reaction time = 60 min).**

Electrochemical Fenton and chemical Fenton processes were compared based on their efficiencies and operational costs and the results are illustrated in Figures 8.

## Discussion

### Effect of electrical current

One of the critical parameters in the electrochemical processes is the electrical current which is responsible for the generation of metal ions within the electrochemical cell. This parameter directly determines the extent of anodic dissolution of iron electrode. In fact, in addition to electrolysis, applied current plays the role of ferrous ion as a catalyst in the electrochemical Fenton process. The correlations of COD removal and decolorization with the applied current are illustrated in Figures 2 and 3. Figure 2 demonstrates COD and color removal within 1483 mg/L of H_2_O_2_ when applied current was varied from 150 to 450 mA. As the applied current increased from 150 to 450 mA, the percentages of decolorization in 30 minutes are 54.6%, 59.8%, 64.5%, and 66.4%, respectively. It was found that removal efficiencies of color and COD were increased with increasing electrical current. This increase is due to that the higher the applied current is, the more ferrous ion can be generated in EF process which in turn, increases the generation of hydroxyl radical. According to Figure 2, in 150 mA and 250 mA, COD removal as a function of time has an upward trend whereas in higher electrical currents (350 mA and 450 mA), this trend decreases slightly after the 45th minute. Similar with the electro Fenton process studied by Atmaca, this decline in efficiency might be due to the gradual dissolution of adsorbed organics from Fe(OH)_n_ flocs prior to the sampling [22]. Figure 3 depicts the efficiencies of COD and color removal in hydrogen peroxide concentration of 2472 mg/L. Generally, by increasing the reaction time, removal efficiencies are promoted; nevertheless, within 350 mA and 450 mA, after 45 minutes of reaction time, there are unexpected fallings in the efficiencies which the reasons were discussed previously. In conformity with literatures, the maximum color removal efficiency can be achieved in a certain ratio of Fe^2+^ (relevant to the electrical current) and H_2_O_2_ concentrations [10].

### Effect of hydrogen peroxide concentration

The H_2_O_2_ concentrations were selected based on stoichiometric weight ratio of the hydrogen peroxide and COD in condition of complete oxidation of COD (R = H_2_O_2_/COD = 2.125) [20]. The selected H_2_O_2_ concentrations including 989, 1483, 1978 and 2472 have weight ratios of 0.55, 0.82, 1.1 and 1.37 respectively. According to Figure 4, which is corresponded to 150 mA electrical current and different concentrations of hydrogen peroxide ranging from 989 to 2472, COD and color removal diagrams versus time have rising trends. Regarding to the Fenton's reaction, the concentration of hydroxyl radical is assumed to increase with increasing H_2_O_2_ concentration [9,26]; but up to a certain amount which endorses the fall of degradation efficiency after increasing the hydrogen peroxide concentration to 2472 mg/L. The highest efficiency was obtained in 1978 mg/L hydrogen peroxide. A sufficient amount of hydrogen peroxide must be present in the system to avoid build up of undesirable intermediates, which is frequently encountered as a major problem during colored wastewater treatment.

As shown in Figure 5, by applying 350 mA electrical current, COD removal and decolorization are improved by increasing the reaction time. Nevertheless, removals of these parameters fall down after 45 minutes. However, these deficiencies in removal do not occur for the sets of experiments that were held within 1978 mg/L hydrogen peroxide. The mentioned decrease might be regarded to the following reasons: As the reaction time increases, the hydrogen peroxide is self-decomposed according to (eq. 4). Moreover, hydrogen peroxide reacts with hydroxyl radicals and acts as scavenger for the hydroxyl radical to produce hydroperoxyl radical which is of less reactivity in comparison with ^•^OH (eq. 5) [14,26]. It is also hypothesized that hydrogen peroxide may be decomposed by the electrolysis within high electrical currents. In 1978 mg/L H_2_O_2_ (R = 1.1), the optimal balance of H_2_O_2_ concentration and electrical current (representing iron dissolution) occurred for the Fenton reaction. The maximum removal efficiencies of 72.9% and 70.6% were obtained for color and COD in conditions of 350 mA (theoretical amount of 1.8 g/L Fe^2+^ based on Faraday's law) [4], 1978 mg/L hydrogen peroxide and 60 minutes reaction time.

(4) $$H_{2}O_{2}+H_{2}O_{2}\to {4H}^{+}+{2O}_{2}+{4e}^{-}$$

(5) $$H_{2}O_{2}{+}^{•}\mathrm{OH}\to {\mathrm{HO}}^{•}{}_{2}+H_{2}O$$

### Chemical Fenton process

Among various ratios of H_2_O_2_ and Fe^2+^ used for the CF process, it was seen that at the ratio of 1978:250 (mg:mg), COD removal and decolorization efficiencies of 51.2% and 52.3% were achieved respectively after 120 minutes of reaction time, which are the highest efficiencies of the CF process in the present study. There are little differences between the decolorization and COD removal efficiencies in all experiments. According to Figures 6 and 7, in spite of increasing Fenton reagents concentrations, degradation rate of organic compounds changes scarcely that might be due to low concentrations of hydroxyl radical in the solution. Moreover, the interference of several dyes and additives in the real wastewater mitigates the efficiency of CF process. Within all experiments, during first 60 minutes of the reaction, degradation efficiencies were inconspicuous; so that COD removal efficiencies were less than 40%. It seems that this wastewater is a complex matrix of refractory organic matters [27]. According to Figure 7, the first points that decolorization efficiencies exceed 50% are attributed to H_2_O_2_:Fe^2+^ of 1978:250 and 2472:350 which are achieved after 120 minutes of reaction time. In fact, it is important to optimize the ratio of H_2_O_2_:Fe^2+^. Not only does the ratio of H_2_O_2_:Fe^2+^ directly affects the production of ^•^OH in Fenton’s reaction, but also increasing ferrous ion is related to the amount of sludge generated from CF process [28,29]. In the present study, the optimum molar ratio of H_2_O_2_:Fe^2+^ for the removal of COD was experimentally detected as 7.9:1.

### Comparison of electrochemical Fenton and chemical Fenton processes

The highest removal efficiencies and operational costs of the two processes are illustrated in Figure 8. Obviously, the EF process efficiency was higher than that of the CF process.

It is also important to consider the experimental conditions in which the processes had their highest efficiencies. The mentioned conditions for the EF were 350 mA, hydrogen peroxide concentration of 1978 mg/L and 60 min reaction time and for the CF process were 250 mg/L Fe^2+^, hydrogen peroxide concentration of 1978 mg/L and 120 min reaction time. It is worth to consider that the maximum removals for both processes were obtained at the hydrogen peroxide concentration of 1978 mg/L. Besides, the highest efficiencies for the CF and EF processes were gained after 120 and 60 minutes respectively. Hence, the reaction time to gain the highest efficiency for the CF process was two times more than that of the EF process which shows that electrochemical processes can significantly reduce the reaction time. Apart from process efficiency, operational costs are also of high concern to evaluate economical feasibility of the process. EF and CF processes were also compared based on their operational costs. In order to economically evaluate the two processes, there are some parameters to be considered. For the EF process, total cost is the sum of costs related to electrical energy consumption, anodic dissolution of iron and amount of hydrogen peroxide consumed. Electrical energy consumption was calculated through equation 6:

(6) E=UIt/kgCOD

Where E is the electrical energy in kWh/kg removed COD, U is the applied voltage (volt), I is the electrical current (A) and t is the reaction time (h). Moreover, theoretical iron dissolution within the EF cell was calculated according to the Faraday’s law [30]:

(7) w=MIt/nF

Where w is the quantity of iron dissolution from anode (g), M is the molecular weight of the iron (g/mol), I is the electrical current (A), t is the reaction time (s), n is the number of electrons and F is the Faraday constant (F = 96487 C/mol). For the CF process, total cost is merely attributed to the amounts of chemicals used (hydrogen peroxide and FeSO_4_.7H_2_O). In this way, the calculated costs for the EF and CF processes were 17.56 and 8.6 US$ per kilogram of the removed COD in Iranian market in July 2013 respectively. At the same time, it is notable that the major part of the EF process costs as an electrochemical process is allocated to the cost of electrical energy consumption. In this study, wastewater sample had negligible electrical conductivity (EC) imposing relatively high resistance to the system which in turn, increased the required applied voltage. Nevertheless, this can be overcome by increasing the EC by adding certain amount of supporting electrolyte. The cost of iron salts in CF process would be reduced by recycling the Fenton sludge. Likewise, in order to minimize the cost of EF process, iron scrap can be used instead of iron sheet as electrode material.

## Conclusions

Electrochemical Fenton and chemical Fenton processes were compared for the degradation of real high strength textile wastewater based on decolorization and COD removal. Applying electrochemical Fenton, the best COD removal and decolorization efficiencies were 70.6% and 72.9% respectively within 350 mA electrical current and 1978 mg/L hydrogen peroxide after 60 minutes. About chemical Fenton process, the highest COD removal and decolorization efficiencies were 51.2% and 52.3% respectively which was achieved in H_2_O_2_:Fe^2+^ ratio of 1978:250 and 120 minutes reaction time. The results show that the electrochemical processes enhance chemical Fenton process and increase the removal efficiency. Last but not least, EF and CF processes were compared based on their calculated operational costs. Though the EF process operational cost was about 2 times more than that of the CF process (17.56 and 8.6 US$ per kilogram of the removed COD for EF and CF processes); it provided higher degradation efficiencies within shorter reaction time. Sludge production and the need for hydrogen peroxide are drawbacks of the CF and EF processes. Besides, electrical energy consumption limits application of EF process economically.

## Competing interests

The authors declare that they have no competing interests.

## Authors’ contributions

All authors have truly been involved within different steps of the current study including conception and design, implementation of the experiments, data collection, analysis, results interpretation and manuscript drafting. Eventually, all authors read and approved the manuscript. All authors read and approved the final manuscript.
